# T cell immunity as a tool for studying epigenetic regulation of cellular differentiation

**DOI:** 10.3389/fgene.2013.00218

**Published:** 2013-11-12

**Authors:** Brendan E. Russ, Julia E. Prier, Sudha Rao, Stephen J. Turner

**Affiliations:** ^1^Department of Microbiology and Immunology, The University of MelbourneParkville, VIC, Australia; ^2^Department of Molecular and Cellular Biology, Canberra UniversityCanberra, ACT, Australia

**Keywords:** epigenetics, T cell Immunity, T cell memory, viral immunity, T cell differentiation

## Abstract

Cellular differentiation is regulated by the strict spatial and temporal control of gene expression. This is achieved, in part, by regulating changes in histone post-translational modifications (PTMs) and DNA methylation that in turn, impact transcriptional activity. Further, histone PTMs and DNA methylation are often propagated faithfully at cell division (termed *epigenetic* propagation), and thus contribute to maintaining cellular identity in the absence of signals driving differentiation. Cardinal features of adaptive T cell immunity include the ability to differentiate in response to infection, resulting in acquisition of immune functions required for pathogen clearance; and the ability to maintain this functional capacity in the long-term, allowing more rapid and effective pathogen elimination following re-infection. These characteristics underpin vaccination strategies by effectively establishing a long-lived T cell population that contributes to an immunologically protective state (termed *immunological memory*). As we discuss in this review, epigenetic mechanisms provide attractive and powerful explanations for key aspects of T cell-mediated immunity – most obviously and notably, immunological memory, because of the capacity of epigenetic circuits to perpetuate cellular identities in the absence of the initial signals that drive differentiation. Indeed, T cell responses to infection are an ideal model system for studying how epigenetic factors shape cellular differentiation and development generally. This review will examine how epigenetic mechanisms regulate T cell function and differentiation, and how these model systems are providing general insights into the epigenetic regulation of gene transcription during cellular differentiation.

## INTRODUCTION

Protection from the myriad of infectious pathogens we are exposed to on a daily basis largely results from the coordinated interaction of the cells and molecules of the mammalian immune system. Key cellular components of the adaptive immune system are white blood cells (*lymphocytes*) of which there are two types: B and T cells. B and T cells share features of adaptive immunity that include the ability to recognize pathogen components via clonal expression of a unique cell surface receptor; the ability to rapidly proliferate upon recognition of a pathogen, coincident with acquisition of cell lineage-specific immune functions; and finally, the ability to persist after the infection is cleared, combined with the capacity to “remember” the pathogen and respond more rapidly and vigorously upon re-infection (termed *immunological memory*).

T cells can be further divided into helper T (T_H_) cells and cytotoxic (killer) T cells. T_H_ cells are distinguished by cell surface expression of CD4 (i.e. CD4^+^ T cells) and promote effective immunity by secreting molecules that promote effective antibody and cellular responses upon infection. Further, T_H_ cells can differentiate into at least six subtypes, each characterized by expression of different immune molecules (termed *effector* molecules), which in turn, dictates that each T_H_ subset can play a different role in immunity to infection. In contrast, killer T cells, distinguished by cell surface expression of CD8 (i.e., CD8^+^ T cells), are the “hit-men” of the immune system, typically locating and destroying virus-infected host cells, and thus limiting and contributing to the eventual clearance of infection. Killer T cells express a range of effector molecules that equip them to mediate this signature killing capacity.

**FIGURE 1:**
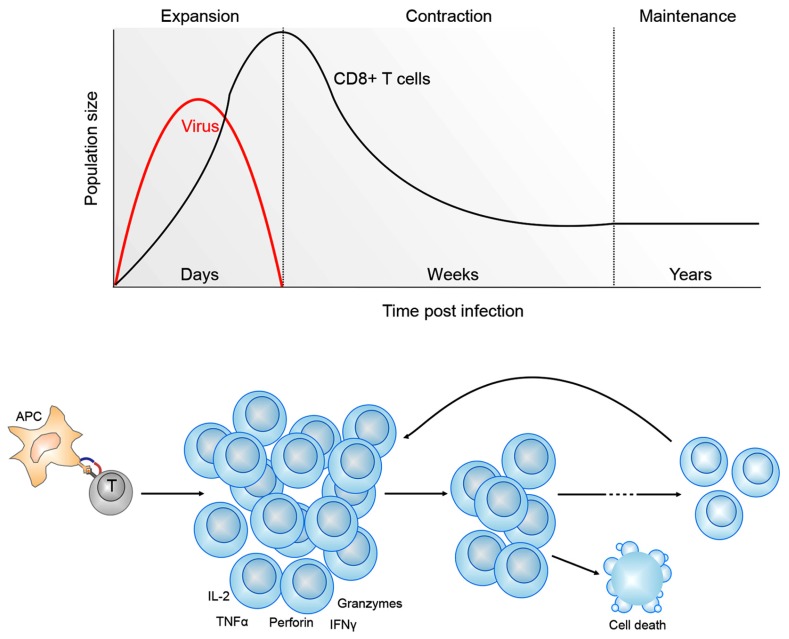
**Kinetics of CD8**^+^ T cell differentiation following viral infection. Shown is a typical CD8^+^ T cell response to a acute viral infection. Antigen presenting cells (APC) present viral antigens to CD8^+^ T cells. This initiates a program of clonal expansion and differentiation into effector CD8^+^ T cells capable of lineage-specific effector functions, including the ability to secrete pro-inflammatory (TNF-α, IFN-γ) and cytotoxic (perforin, granzyme) molecules. Following viral clearance, the CD8^+^ T cells undergo an extensive contraction phase, mediated by programed cell death. The remaining memory CD8^+^ T cells can persist in the host for years. In the event of a secondary exposure to the same virus, memory CD8^+^ T cells can rapidly expand and acquire effector functions.

A cardinal feature of T cell immunity is the ability of naïve T cells to undergo a program of proliferation and functional differentiation upon activation, resulting in a large pool of cells, all capable of recognizing a particular pathogen, and that have acquired the immune functions necessary to control and eventually clear infection (Kaech et al., 2002; van Stipdonk et al., 2003; **Figure 1**). Once an infection is cleared, the majority of the expanded effector T cell population dies, leaving behind a small pool of long-lived cells that can recognize the same pathogen that triggered their initial activation (termed memory T cells; Marshall et al., 2001; Kaech et al., 2002; La Gruta et al., 2004). Importantly, these memory T cells produce a broader array of immune molecules than naïve cells, and in larger quantities, and unlike naïve cells, can respond to infection without the need for further differentiation (Lalvani et al., 1997; Agarwal and Rao, 1998; Oehen and Brduscha-Riem, 1998; Veiga-Fernandes et al., 2000). These features, combined with persistence at a higher frequency, enable memory T cells to respond more rapidly upon secondary infection, enabling earlier control and clearance of infection (**Figure 1**), and together, these features of memory T cells provide the basis of T cell-mediated immunity. Importantly, our understanding of the molecular factors that shape cell fate decisions and drive acquisition of T cell effector function is limited, and questions remaining to be determined include how a T cell decides to be a memory versus an effector cell, and what are the molecular mechanisms that enable stable maintenance of rapid effector function within memory T cells in the long-term? In this review we describe what we think are some of the more interesting and important studies addressing these and similar questions, with the aim of demonstrating the utility of the immune system as a tool for studying epigenetics and cellular differentiation. We start by discussing the diversity of T cells phenotypes, before describing our current understanding of how epigenetic regulation influences how these distinct functional T cell populations arise and are maintained.

## DEFINING THE DIFFERING ROLES OF DISTINCT T CELL SUBSETS IN MEDIATING IMMUNITY

**FIGURE 2:**
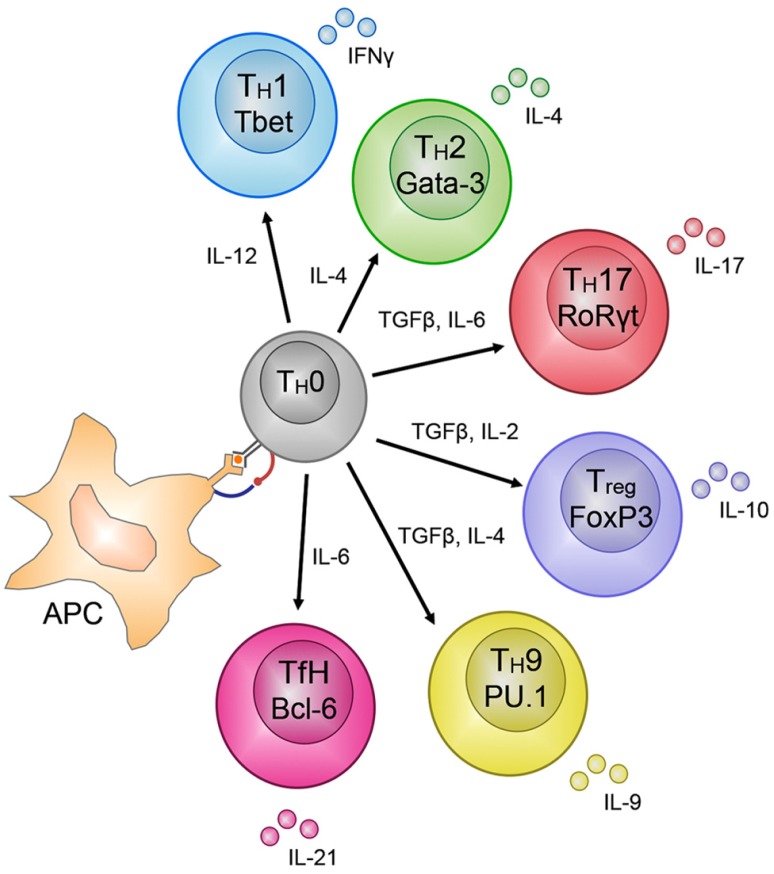
**CD4**^+^ T_**H**_ – cell subset differentiation. CD4^+^ T cells show remarkable plasticity and are able to differentiate into many different subsets based on the soluble molecules secreted during priming of the subsets by antigen presenting cells (APC), e.g., IL-12 for T_H_1 cells. The different subsets can be distinguished by the transcription factors that regulate and maintain their lineage-specific effector functions, e.g., T-bet for T_H_1 cells. The molecules secreted by these subsets, e.g., IFN-γ for T_H_1 cells, are finely tuned to control the pathogen that mediated the release of the specific molecules by the APC during activation of the T_H_0 cells into the various subsets.

An important feature of T cell immunity is the enormous proliferative potential and functional plasticity of naïve T cells. Acquisition of lineage-specific T cell effector functions is clearly linked to an extended proliferative response, suggesting that T cell activation engages a differentiation program that facilitates effector gene expression (Gett and Hodgkin, 1998; Lawrence and Braciale, 2004; Jenkins et al., 2008). An example of T cell functional plasticity is found after activation of naïve T_H_ cells that have the potential to differentiate into distinct T cell subsets, largely defined by the soluble effector molecules they secrete (**Figure 2**; Zhu et al., 2010). The best characterized of these are the T_H_1 and T_H_2 subsets, however, other subsets include T_H_17, Tregs (regulatory T cells), T_FH_ (follicular T_H_ cells) and the more recently described T_H_9 cells (**Figure 2**). T_H_1 and T_H_2 T cells are best characterized by their capacity to secrete interferon-gamma (IFN-γ) and interleukin (IL)-4, respectively. The tailoring of T_H_ cell responses into distinct functional lineages is a consequence of integration of multiple signals that are present during initial T cell activation (**Figure 2**). For example, naïve T_H_ cell activation in the presence of the pro-inflammatory molecules, IFN-γ and IL-12, induces T_H_1 differentiation while IL-4 is a potent inducer of T_H_2 differentiation (Zhu et al., 2010). Importantly, induction of transcription factor (TF) expression by extracellular signals received by activated T_H_ cells drives T cell differentiation (Kanno et al., 2012); T_H_1 differentiation is dependent on STAT1 activation and expression of the T-box TF *Tbx21* (T-bet; Djuretic et al., 2007). Conversely, IL-4 signals activate STAT6 resulting in up-regulation of the TF *Gata3* (Ansel et al., 2003). T_H_17 differentiation is associated with IL-6/IL-21 induced expression of the RORγT TF (Dong, 2008) and Treg differentiation with FoxP3 (reviewed in Josefowicz et al., 2012). Such is the importance of these TFs in directing naïve T_H_ cell commitment to a specific lineage that they are used as definitive markers of T_H_ subset differentiation (**Figure 2**).

As we learn more about these T_H_ subsets, it is clear that there is heterogeneity of effector function within a responding T cell population such that no one immune response is uniquely represented by a single T_H_ subset. Rather, there is tailoring of the total T cell population such that a particular subset may be over-represented. For example, T_H_1 type cells dominate the response to extracellular bacterial infections, and in this case, expression of the T_H_1 cytokine, IFN-γ, is required to promote immune control of these particular pathogens. In this way, the immune system ensures that the most appropriate immune response is engaged to promote control of infection.

Killer T cells contribute to the control and eventual elimination of intracellular bacteria, viruses and tumor challenges via the coordinated interplay of varied effector mechanisms (Russ et al., 2012). This includes the production of pro-inflammatory cytokines such as IFN-γ and tumor necrosis factor alpha (TNF-α; La Gruta et al., 2004) and the expression of cytolytic effector molecules including perforin (Pfp; Kagi et al., 1994) and the granule enzymes (granzymes, Gzm) A, B, and K (Jenkins et al., 2007; Peixoto et al., 2007; Moffat et al., 2009). Whilst killer T cells are not typically associated with commitment to distinct lineages, it is clear that specific TFs are also important in regulating their differentiation and acquisition of effector function. For instance, two T-box TFs, T-bet (encoded by *Tbx21*) and Eomesodermin (encoded by *Eomes*; Intlekofer et al., 2005) play essential roles in effector CTL differentiation. Analogous to its role in T_H_1 T cells, T-bet is rapidly up-regulated upon naïve killer T cell activation and directly regulates the rapid acquisition of IFN-γ production (Cruz-Guilloty et al., 2009). Eomesodermin, a homolog of T-bet, was originally implicated in the regulation of CD8^+^ T cell granzyme B expression (Pearce et al., 2003), however, recent studies suggest that Eomesodermin is expressed later during CTL differentiation and contributes more to acquisition of perforin expression, while helping sustain the capacity to express IFN-γ (Cruz-Guilloty et al., 2009). IL-2 is a cytokine required for inducing proliferation and survival of activated T cells (Miyazaki et al., 1995). Importantly, high levels of IL-2 signaling at the time of killer T cell activation contribute to granzyme B and perforin expression via STAT5 activation (Janas et al., 2005; Pipkin et al., 2010). In this way, killer T cells integrate signals delivered by extrinsic inflammatory and survival signals during infection that promote effector T cell differentiation.

While the importance of these TFs in lineage determination is clear, exactly how they convey their effects on T cell differentiation is less well understood. As we describe below, at least some of these TFs (i.e., STAT6 and T-bet) exert their effects on T cell differentiation through the recruitment of chromatin modifying enzymes to the sites of TF binding (Lewis et al., 2007; Miller et al., 2010; Onodera et al., 2010). Further, such mechanisms of TF action are known from other systems, suggesting that this mechanism may be common. Thus it appears that TFs and chromatin modifying enzymes cooperate, with the former providing the DNA binding specificity, and the latter the catalytic activity. As described below, once modified, the chromatin can then serve as a substrate for yet other protein complexes that physically rearrange the chromatin, making it more or less permissive for transcription.

## EPIGENETIC REGULATION OF CELLULAR DIFFERENTIATION

Cellular differentiation is regulated by the strict spatial and temporal control of gene expression, which at the most fundamental level, is controlled by modulating access of the transcriptional machinery to gene regulatory regions, including promoters and enhancers. In eukaryotic cells, transcription occurs in the context of chromatin – a complex formed between the genome and histone protein octomers (termed nucleosomes), around which the DNA is wound. As the intimate nature of the nucleosome–DNA interaction can occlude binding of the transcriptional machinery, preventing transcription, this interaction must be tightly regulated to allow appropriate gene expression; this is achieved by controlling the positioning of nucleosomes, and by modulating their affinity for DNA. Histone post-translational modifications (PTMs) are key regulators of changes in chromatin structure that then influence gene expression. Importantly, these modifications are often propagated faithfully at cell division (termed *epigenetic* propagation), maintaining cellular identity in the absence of signals driving cellular differentiation.

Histone PTMs occur primarily at the solvent exposed N-termini, and can take a number of forms, including acetylation, methylation, and ubiquitination (Kouzarides, 2007). The transcriptional consequences of these modifications are then manifested either due to the direct biophysical consequences of the modification, or through the catalytic activities of proteins and protein complexes that recognize and bind modified histones. For instance, acetylation, which reduces the net positive charge on the nucleosome, results in decreased stability of histone associations with the negatively charged DNA, promoting transcription. Therefore, by balancing the expression and genomic localization of histone acetyltransferases (HATs) and histone deacetylases (HDACs), which add and remove acetyl groups, respectively, transcription can be activated or repressed (reviewed in Bannister and Kouzarides, 2011). Alternatively, it appears that the effects of histone methylation are conveyed indirectly, with methylated histones serving as a substrate for protein complexes that bind and reconfigure the chromatin. Importantly, histone methylation is associated with both active and repressed transcription, depending on the residue methylated. For example, trimethylation of lysine 4 of histone H3 (H3K4me3) is enriched at promoters of many actively transcribed genes, while trimethylation of lysine 27 of H3 (H3K27me3) is associated with transcriptionally repressed genes (Barski et al., 2007; Wang et al., 2008; Ernst et al., 2011).

Interestingly, activating and repressive modifications can co-localize, even occurring on the same nucleosome, and it appears that the combination and balance of these modifications serves to tune levels of transcription (Wang et al., 2008). Importantly in the context of cellular differentiation, co-localization of opposing PTMs is also employed to poise genes for rapid activation or repression (Bernstein et al., 2006).

As well as controlling access of the transcriptional machinery to the DNA template by modulating nucleosome positioning, transcription is controlled epigenetically by changing the structure of the DNA itself, through the addition and removal of bulky methyl groups. DNA methylation occurs predominantly at cytosine residues occurring in the context of cytosine–guanine di-nucleotides (termed *CpG methylation*), and results in transcriptional repression, both through steric hindrance of transcriptional activator binding (as described below for FoxP3), and through recruitment of methyl-CpG-binding domain proteins (MBD), that in turn, recruit HDACs. For instance, MBD2 has been shown to directly recruit HDAC1, resulting in histone deacetylation, and transcriptional repression (Ng and Bird, 1999). Thus, CpG methylation does not represent a separate system of epigenetic regulation to that described for histone PTMs, but rather is part of the same, inter-connected system.

## EPIGENETIC CONTROL OF CD8^+^ T CELL EFFECTOR FUNCTION

The function of CD8^+^ killer T cells is defined largely by their capacity to produce effector molecules such as anti-viral cytokines and cytolytic molecules. As with naïve T_H_ cells, the *Ifng* locus of naïve CD8^+^ killer T cells is heavily marked by the repressive H3K27me3, with little or none of the permissive H3K9Ac or H3K4me3 PTMs (Denton et al., 2011). Upon differentiation from naïve to effector killer T cells, transcriptional activation of *Ifng* is associated with removal of H3K27me3 and deposition of the permissive H3K9Ac and H3K4me3 PTMs (Denton et al., 2011). Further, in effector CD8^+^ killer T cells, the *Ifng* locus had reduced levels of total histone H3, indicating nucleosome evacuation from the region, presumably to allow the transcriptional machinery to access the promoter. Taken together, these data suggest that reconfiguration of the chromatin structure within naïve cells is necessary to enable *Ifng* transcription. Moreover, there appears to be conservation of chromatin restructuring and histone PTM modification with a similar pattern observed within other effector gene loci such as granzyme B (*Gzmb*; Juelich et al., 2009) and granzyme A (*Gzma*; Lauren Hatton, Michelle Nguyen, Brendan Russ, and Stephen Turner, data not shown).

**FIGURE 3:**
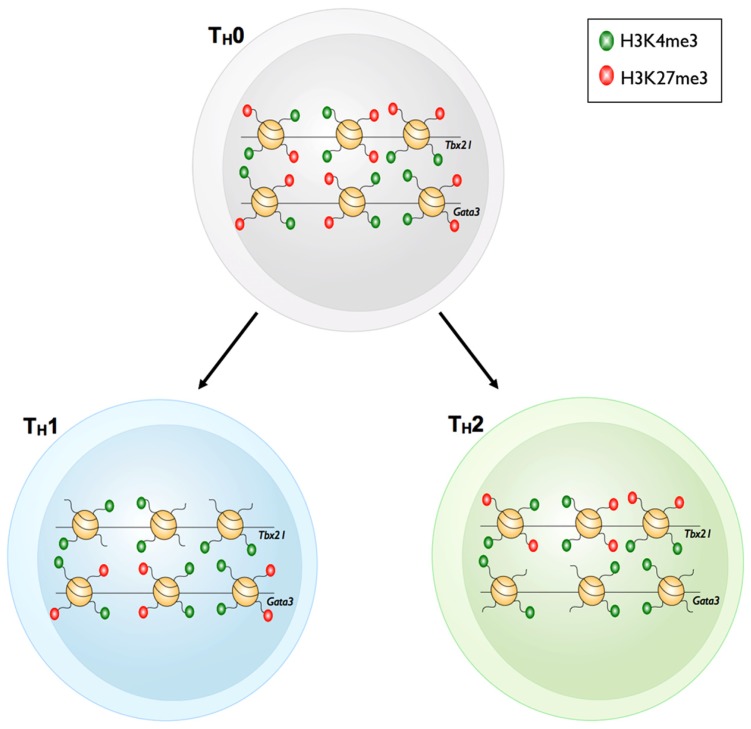
**Bivalency of master regulator gene loci in T_H_1 and T_**H**_2 cells**. Prior to differentiation of T_H_0 cells into the T_H_1 or T_H_2 cell subsets, the master regulator loci of each subset, *Tbx21* and *Gata3* respectively, have both active H3K4me3 and repressive H3K27me3 marks. The bivalent *Tbx21* locus loses the repressive H3K27me3 mark upon differentiation into T_H_1 cells. However, the *Gata3* locus remains bivalent. The reverse is true for T_H_2 cells whereby *Gata3* loses the repressive H3K27me3 mark yet retains bivalency at the *Tbx21* locus.

As mentioned earlier, memory T cells maintain the capacity for rapid effector gene expression without the need for further differentiation. Strikingly, the permissive signature within the *Ifng* promoter of effector CD8^+^ killer T cells is maintained into long-term memory. Further, although memory CD8^+^ killer T cells exhibit little *Ifng* transcriptional activity prior to re-infection, RNA polymerase (RNAp) is docked at the *Ifng* promoter (Denton et al., 2011; Zediak et al., 2011). Taken together, these data suggest that the ability of memory cells to produce IFN-γ rapidly following re-infection is due to the promoter being maintained in a transcriptionally permissive state, and that the rate-limiting step in re-expression of IFN-γ is transcriptional initiation (**Figures 3A,B**). It remains to be determined whether transcriptional poising (as measured by RNAp docking) at other effector gene loci with low transcriptionally activity is evident within memory CD8^+^ killer T cells. Further, it would be of particular interest to determine the extent of transcriptional poising in memory T cells at a genome-wide level and compare this to naïve and effector cells. In this way, it could determined to what extent transcriptional poising underpins memory T cell characteristics. Moreover, given the direct effect of acetylation on nucleosome density, increased acetylation in memory cells (Araki et al., 2008; Denton et al., 2011) may explain their ability to produce more IFN-γ upon re-infection (La Gruta et al., 2004). In this way, memory T cells are reconfigured at the chromatin level to exhibit more potent effector function and this, in turn, helps ensure more effective and more rapid control of a secondary infection.

Recently, Scharer et al. (2013) applied global approaches to compare CpG methylation in naïve and effector CD8^+^ T cells. Combining immunoprecipitation of methylated genomic regions with high-throughput sequencing (MeDIP-seq), they identified ~650,000 regions that were differentially methylated between the two populations, indicating the likely importance of CpG methylation as a means of regulating CD8^+^ T cell differentiation. As expected, CpG methylation of gene promoters was inversely correlated with gene transcription, but interestingly, ~40% of genomic regions that differed in methylation state between naïve and effector occurred away from gene promoters. Further analysis showed that these promoter-distal regions largely overlapped candidate transcriptional enhancers identified in developing T cells in the thymus using next-generation sequencing and chromatin immunoprecipitation (ChIP-Seq) for enhancer-enriched histone PTMs (H3K27Ac and H3K4me1). Finally, when these putative enhancers were surveyed for over-represented TF binding sites, known and putative transcriptional regulators of CD8^+^ T cell differentiation were identified. Therefore, it seems likely that CpG methylation is employed to regulate CD8^+^ T cell differentiation, both by influencing protein–DNA interactions at gene promoters, and at transcriptional enhancers. Further, this study highlights the utility of such approaches in the identification of regulatory circuits controlling cellular differentiation.

## CD4^+^ T CELL DIFFERENTIATION: A MODEL FOR UNDERSTANDING EPIGENETIC REGULATION

The fact that distinct signals are capable of driving naïve T_H_ cell differentiation *in vitro* into well-defined subsets makes CD4^+^ T cell activation a useful model for understanding how epigenetic regulation can influence cellular differentiation and fate determination. Comparison of the epigenetic profiles of signature effector gene loci within T_H_1 and T_H_2 cells has been particularly informative. In response to T_H_1 differentiation signals, the IFN-γ locus of naïve T_H_ cells is remodeled to a permissive epigenetic signature that reinforces and heritably maintains IFN-γ gene expression in the long-term. At the same time, the IL-4 locus is remodeled to have a repressive epigenetic signature resulting in the shutdown of IL-4 gene expression.

Recent work using ChIP-Seq has been instrumental in providing genome-level insights into how epigenetic processes might regulate T_H_ cell fate selection. For instance, genome-wide comparison of H3K4me3 and H3K27me3 distribution in naïve, T_H_1, T_H_2, and T_H_17 cells, combined with global transcriptional profiling demonstrated that the distribution of just two histone PTMs (H3K27me3 and H3K4me3) could provide a simple explanation for the differences in phenotypes observed amongst these different T cell subsets.

**FIGURE 4:**
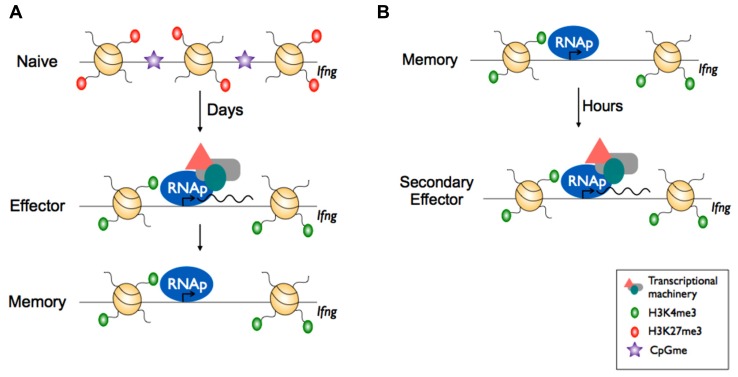
**Epigenetic reprogramming within effector gene loci of CD8^+^ memory T cells enables rapid effector function.** **(A)** In naïve CD8^+^ T cells, effector loci such as *Ifng* display repressive epigenetic marks e.g., H3K27me3 and is inaccessible to transcriptional machinery due to the heterochromatin structure. Upon activation, the chromatin is remodelled whereby it acquires active epigenetic marks e.g., H3K4me3 at key effector loci and nucleosome exit to make the loci accessible by transcriptional machinery and RNA polymerase II (RNAp), allowing transcription. Upon differentiation to memory CD8^+^ T cells, the chromatin retains the permissive H3K4me3 mark and RNAp remains docked. **(B)** Upon re-infection, the effector loci in memory CD8^+^ T cells is poised and can undergo rapid transcription.

For example, upon differentiation from a naïve T_H_ state into the various T_H_ subsets, H3K4me3 deposition was observed at signature effector gene loci within distinct T_H_ subsets (e.g., *Ifng* in T_H_1, *Il4* in T_H_2, and *Il17* in T_H_17). Moreover, H3K27me3 deposition was correlated with transcriptional shutdown of effector gene loci that are characteristic of other T_H_ subsets (Wei et al., 2009; **Table 1**). One might have expected that changes in the epigenetic signatures within gene loci encoding lineage-defining TFs, would simply reflect those observed for lineage-specific effector gene loci. For example, the gene locus encoding the T_H_17 TF *Rorc* (retinoid-related orphan receptor-γ) was decorated with H3K27me3 in the naïve state, and only acquired H3K4me3, and losing H3K27me3 after T_H_17 differentiation. In contrast, the repressive H3K27me3 signature was reinforced under T_H_1 and T_H_2 differentiation conditions (Araki et al., 2009). However, this was not always the case. The *Tbx21* (T_H_1) and *Gata3* (T_H_2) gene loci in naïve T_H_ cells were marked with both H3K4me3 and H3K27me3 (termed bivalent loci), and whilst these loci resolved to a permissive epigenetic signature (H3K4me3^+^/H3K27me3^-^) under T_H_1 and T_H_2 differentiation conditions, respectively, they did not acquire a repressive epigenetic signature when differentiated into opposing lineages, but rather maintained a bivalent state (**Figure 4**). Similarly, the *Tbx21* locus within T_H_17 cells was also maintained in a bivalent state. In the case of T_H_17 cells, re-stimulation of T_H_17 cells in the presence of IL-12 resulted in expression of IFN-γ and conversion to a T_H_1 phenotype. This was associated with acquisition of permissive epigenetic signatures at the IFN-γ locus and IL-12-dependent STAT-4 and *Tbx21*-dependent epigenetic silencing of the T_H_17 associated *Rorc* locus (Mukasa et al., 2010). Given that epigenetic bivalency is considered a mechanism for poising gene loci for rapid activation or repression, these data suggest that CD4^+^ T_H_ subsets can maintain some level of functional plasticity despite lineage commitment. It is tempting to speculate that this provides the immune system with inherent flexibility, allowing the redirection of pathogen-specific T_H_ responses. In the case of T_H_17 cells, it may represent a mechanism that enables switching from a potent inflammatory T_H_17 response to a less damaging, more controlled effector response. It also suggests that targeted interventions that drive epigenetic reprograming of T_H_ responses involved in autoimmune diseases (such as T_H_17 in the context of multiple sclerosis) might represent novel immunotherapeutic targets that could lead to decreased pathology.

**Table 1:** Major histone methylation patterns at lineage-specific effector gene loci in differentiated CD4^+^ T_**H**_ populations.

	**T**_**H**_1	**T**_**H**_2	**T**_**H**_17
*Ifng*	H3K4me3^+^	H3K27me3^+^	H3K27me3^+^
*Il4*	H3K27me3^+^	H3K4me3^+^	H3K27me3^+^
*Il17a*	H3K27me3^+^	H3K27me3^+^	H3K4me3^+^

A number of studies have also defined roles for CpG methylation in the differentiation of CD4^+^ T cells. For instance, regulated deposition of CpG methylation is important for maintenance of CD4^+^ T cells that have differentiated to become Tregs. Zheng et al. (2010) showed that mice that had a conserved non-coding sequence (CNS2) within the *Foxp3* locus deleted, had wild-type levels of Tregs in young mice, but greatly reduced numbers in older mice. Further, this was due to a loss of FoxP3 expression in the peripheral Tregs, indicating a role for this TF, not just in Treg differentiation, as described previously (reviewed in Josefowicz et al., 2012), but also in the maintenance of the Treg phenotype. Finally, they were able to show that FoxP3 binds to the CNS2 in Tregs, but not in naïve CD4^+^ T cells, and that FoxP3 binding was dependent on differentiation-induced demethylation of CpG sites within this region. Thus, FoxP3 binding to CNS2, enabled by differentiation-dependent CpG demethylation, results in a feed-forward signal that enforces Treg fate.

## ENZYMES MODULATING HISTONE MODIFICATION DURING T CELL DIFFERENTIATION

Whilst there is a growing understanding of how changes in histone PTMs correlate with dynamic changes in T cell effector functions, it is less clear how the factors that write or erase these histone PTMs are involved in directing T cell differentiation during an immune response. Using the CD4 T_H_1 versus T_H_2 model system, Allan et al. (2012) examined the role of the histone methyltransferase, Suv39H1, in epigenetic regulation of T_H_2 differentiation. Suv39H1 specifically trimethylates H3K9 – a PTM typically associated with transcriptional silencing of gene loci that is in turn recognized by heterochromatin protein 1α (HP1α; Lachner et al., 2001; Peters et al., 2001). Docking of HP1α onto H3K9me3^+^ gene loci in turn recruits HDAC1 and 2, and the transcriptional repressor MBD1 (Fujita et al., 2003). In this way, H3K9 acetylation, a PTM associated with transcriptional activation, is limited. Thus, Suv39H1-mediated trimethylation of H3K9 is an initial step that triggers histone deacetylation and binding of transcriptional repressor protein complexes that stably silence targeted loci.

While it was possible to skew naïve T_H_ cells from Suv39H1 gene-deficient mice into the T_H_2 lineage *in vitro*, these cells could be reprogrammed to secrete IFN-γ after re-culture in T_H_1-inducing conditions. Thus, a lack of Suv39H1 resulted in an inability to stably repress T_H_1 effector gene expression. This appeared largely due to an inability of Suv39H1 gene-deficient T_H_2 cells to stably silence the transcriptional potential of the key T_H_1 TF, T-bet (encoded by *Tbx21*). Consistent with this, Suv39H1-deficient T_H_2 cells exhibited increased levels of histone acetylation at the *Tbx21* locus. Of particular interest was the fact that T_H_1 cells from Suv39H1 gene-deficient mice stably repressed expression of T_H_2 effector genes after re-culture in T_H_2-inducing conditions. This suggests that histone PTMs, other than H3K9me3, are used to heritably silence T_H_2 effector gene expression during T_H_1 differentiation, or alternatively, other H3K9 methyltransferases (such as GP9a, SETDB1/2, or Suv39H2) are utilized by T_H_1 cells to establish H3K9me3 repression at T_H_2 gene loci. Such a hypothesis would require selective targeting of H3K9 methyltransferases to specific gene loci and this could potentially be facilitated via interactions with specific TFs that bind to specific regulatory regions within target gene loci. Such a precedent has been observed with the demonstration that members of the T-box family of TFs serve to recruit histone methyltransferases to signature effector gene loci within T_H_1 cells to promote gene transcription (Lewis et al., 2007). Thus, this mechanism could potentially be a way of ensuring that only certain gene loci are targeted for silencing within either T_H_1 or T_H_2 cell subsets, thereby ensuring appropriate gene expression, and appropriate immune function.

**FIGURE 5:**
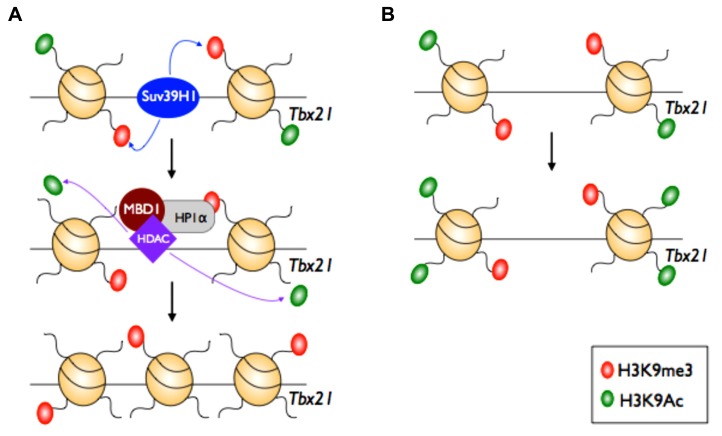
**Epigenetic maintenance of T_**H**_2 lineage commitment.** In the T_H_2 cell subset, the master regulator of T_H_1 cells (*Tbx21*) is silenced. The histone methylase Suv39H1 adds the repressive H3K9me3 mark at the *Tbx21* locus. This initiates recruitment and docking of heterochromatin protein 1 alpha (HP1α), histone deacetylase (HDAC) 1 and 2, and methyl-binding domain protein (MBD1). HDACs then remove the active H3K9ac mark to maintain silencing, mediated by H3K9me3, at the *Tbx21* locus.

Taken together, these data demonstrate that Suv39H1 acts to specifically promote T_H_2 lineage commitment via epigenetic silencing (via H3K9me3 deposition) of gene loci that drive T_H_1 fate commitment (**Figure 5**). One interesting observation was the fact that despite T_H_2 cells exhibiting an overall repressive signature within the *Tbx21* locus, there is still evidence of H3K4me3 deposition at the promoter. Thus, pharmacological interventions that block Suv39H1 activity could serve to promote *Tbx21* transcription and subsequent T_H_1 gene expression. The clinical relevance was made apparent when treatment of mice with a Suv39H1 inhibitor, was able to ameliorate T_H_2 cell driven tissue damage in a model of allergic asthma. Treatment of mice resulted in higher numbers of T_H_1 T cells, and redirected the immune response toward a less pathogenic state. This study highlights the potential for manipulating epigenetic programing of effector T cell responses using small molecule inhibitors to either promote immunity, in the case of vaccination, or suppress the damage caused by inappropriate immune responses, as is found in autoimmune disease or allergy.

## EPIGENETIC CONTROL OF T CELL DEVELOPMENT

Mature, immunologically naïve CD4^+^ and CD8^+^ T cells develop in the thymus from multipotent hematopoietic progenitors. Within the thymus, these progenitors progress through at least ten phenotypically distinct stages of development, before exiting the thymus as mature, naïve CD4^+^ or CD8^+^ T cells (reviewed in Rothenberg et al., 2010). Hematopoietic progenitor cells enter the thymus expressing neither CD4 nor CD8, and are hence termed double-negative (DN). They then progress through five phenotypically distinct stages of maturation (DN1, DN2a, DN2b, DN3a, and DN3b) before up-regulating both CD4 and CD8 (termed *double-positive*, DP), and following further differentiation, permanently down-regulate either CD4 or CD8 (becoming *single-positive*, SP), before migrating from the thymus. Importantly, events occurring in the thymus not only determine lineage commitment (CD4^+^ versus CD8^+^), but also the potential fates of mature T cells; commitment to the CD8^+^ lineage results in cells with specialized cytotoxic potential, while commitment to the CD4^+^ lineage results in naïve cells with much broader differentiation potential. Thus an interesting question is when is fate potential programed, and what is the contribution of epigenetic mechanisms?

Rothenberg’s group recently studied the molecular signatures that underpin lineage commitment and differentiation occurring in the early phases (DN1–DP) of thymic development in mice (Zhang et al., 2012). Combining ChIP-Seq and RNA-Seq, they determined the global distribution and dynamics of three histone PTMs, and the transcriptional signatures of immature thymocytes, at each stage of differentiation. Specifically, they studied the distribution of H3K9/14Ac (Gett and Hodgkin, 1998; Djuretic et al., 2007) and H3K27me3, which is enriched within the promoters and enhancers of actively transcribed and repressed genes, respectively, and H3K4me2, which defines active enhancer elements, and is often associated with transcriptionally poised gene promoters.

Aside from highlighting the extraordinary complexity of the mechanisms regulating T cell differentiation, this study provided novel insights into the mechanisms controlling cellular differentiation. A key finding of the paper was that the repressive H3K27me3 PTM is often deposited at genes after transcription has already been shutdown, indicating that the likely role of this modification is not to directly regulate transcription, as is generally accepted, but rather to stabilize repression. Further, there appeared to be multiple mechanisms of transcriptional repression, since only approximately a third of genes that were developmentally repressed during thymic differentiation were associated with H3K27me3.

In contrast, histone acetylation was strongly and temporally correlated with mRNA levels, indicating that this modification may be added just prior to transcription, and as such, likely represents a rate-limiting step in the activation of gene transcription. Further histone deacetylation may be a key means of gene repression during T cell differentiation since this observation also implies that acetylation is either rapidly removed from promoters following transcriptional repression, or is a direct cause of transcriptional repression. Finally, H3K4me2 deposition often preceded transcription. This finding is consistent with a previous study showing that H3K4me2 marks lineage-specific hematopoietic genes in multipotent progenitor cells, in the absence of transcription (Orford et al., 2008). As many H3K4me2-marked genes lost this modification as differentiation preceded (toward an erythroid fate), it appears that H3K4me2 poises genes for a rapid response to differentiation signals, whereby, following the receipt of signals, non-lineage-specific genes lose H3K4me2 and are not expressed, while at lineage-specific genes, H3K4me2 is converted to H3K4me3 – a positive correlate of transcription.

Taken together, these studies suggest that different histone PTMs play distinct roles in transcriptional regulation; acetylated histones appear to rate-limit transcription, probably by directly regulating promoter accessibility, while H3K27me3 appears to operate “after the fact” – stabilizing transcriptional repression rather than directly repressing transcription. Finally, H3K4me2 apparently functions as an intermediate between unmethylated H3K4, and the activating trimethylated state at gene promoters, thus allowing rapid transcriptional change following differentiation signals.

In the context of T cell development, CpG methylation plays important roles both during thymic development, and in later (peripheral) fate decisions (described above). For instance, by deleting DNA methyltransferase 1 (DNMT1) at the DN stage of thymic development, Lee et al. (2001) showed an ~90% reduction in the numbers of DP T cells, as well as large decreases in mature peripheral T cells of both CD4^+^ and CD8^+^ lineages. Further, the T cells that did develop had greatly reduced survival relative to the wild-type. However, when DNMT1 was deleted at the (later) DP stage, peripheral T cell numbers and composition were normal, but when either (Dnmt1^-^^/^^-^) CD4^+^ or CD8^+^ T cells where stimulated *in vitro*, they had aberrant cytokine production profiles, in that they produced IL-2, IL-3, and IFN-γ more rapidly than wild-type cells. This latter observation is consistent with the demonstration that demethylation of regions controlling the transcription of *Ifng* and *Il2* in effector CD8^+^ T cells (Kersh et al., 2006; Northrop et al., 2006) and *Il2* in effector CD4^+^ T cells (Thomas et al., 2005) coincides with their demethylation. Further, it suggests that methylation might be a safeguard against inappropriate expression of these genes, which might otherwise lead to immune pathology. Taken together, these results indicated a central role for DNMT1, and CpG methylation, both during thymic and post-thymic development and differentiation of T cells of both CD4^+^ and CD8^+^ lineages.

## SUMMARY

Both current effective vaccine strategies, and the design of novel vaccine strategies that specifically target adaptive T cell immunity, rely on acquisition and maintenance of T cell functional potential to establish protective immunity. Conversely, these same characteristics of adaptive T cell immunity are also at play during adverse immune reactions where priming of T cells to either environmental or self-antigens, can manifest as T cell hypersensitivities or T cell-mediated autoimmune diseases, respectively. Thus, a greater understanding of the molecular mechanisms, and specifically epigenetic mechanisms, that shape acquisition and maintenance of lineage-specific T cell function, will be key if we are to make advances in novel therapeutic strategies for a variety of disease contexts. We have tried to highlight what we think are some of the key findings and general themes emerging from the studies of T cell differentiation, as well as the utility of the immune system as a tool for studying differentiation and development. By comparison with studies performed on stem cells, it appears that conclusions made from studies of T cells are broadly relevant to differentiation in other cell types and tissues. In particular, the concepts of transcriptional poising and promoter bivalency as mechanisms that regulate fate decisions are pertinent during the differentiation of stem cells and less primitive tissues. The studies of Rothenberg et al. (2010), in particular, highlight the value of the immune system as a tool for studying differentiation – because of the detailed ontogenies and the ability to resolve different stages of T cell development based on characteristic and defined cell surface phenotypes.

## Conflict of Interest Statement

The authors declare that the research was conducted in the absence of any commercial or financial relationships that could be construed as a potential conflict of interest.
